# Molecular characteristic of treatment failure clinical isolates of *Leishmania major*

**DOI:** 10.7717/peerj.10969

**Published:** 2021-03-11

**Authors:** Gilda Eslami, Samira Hatefi, Vahid Ramezani, Masoud Tohidfar, Tatyana V. Churkina, Yuriy L. Orlov, Saeedeh Sadat Hosseini, Mohammad Javad Boozhmehrani, Mahmood Vakili

**Affiliations:** 1 Department of Parasitology and Mycology, School of Medicine, Shahid Sadoughi University of Medical Sciences, Yazd, Iran; 2 Research Center for Food Hygiene and Safety, School of Public Health, Shahid Sadoughi University of Medical Sciences, Yazd, Iran; 3 Department of Pharmaceutics, School of Pharmacy, Shahid Sadoughi University of Medical Sciences, Yazd, Iran; 4 Pharmaceutical Research Center, School of Pharmacy, Shahid Sadoughi University of Medical Sciences, Yazd, Iran; 5 Department of Biotechnology, Faculty of Life Science and Biotechnology, Shahid Beheshti University, Tehran, Iran; 6 Insitute of Cytology and Genetics SB RAS, Novosibirsk, Russia; 7 Novosibirsk State University, Novosibirsk, Russia; 8 The Digital Health Institute, I.M.Sechenov First Moscow State Medical University (Sechenov University), Moscow, Russia; 9 Department of Community and Preventive Medicine, Health Monitoring Research Center, School of Medicine, Shahid Sadoughi University of Medical Sciences, Yazd, Iran

**Keywords:** Leishmania, Parasitology, Leishmaniasis, Clinical isolates, Minicircle kDNA, COXII, Treatment failure, Phylogenetic analysis, Gene expression

## Abstract

**Background:**

Leishmaniasis is a prevalent tropical disease caused by more than 20 *Leishmania* species (Protozoa, Kinetoplastida and Trypanosomatidae). Among different clinical forms of the disease, cutaneous leishmaniasis is the most common form, with an annual 0.6–1 million new cases reported worldwide. This disease’s standard treatment is pentavalent antimonial (Sb^V^) that have been used successfully since the first half of the 20th century as a first-line drug. However, treatment failure is an increasing problem that is persistently reported from endemic areas. It is important to define and standardize tests for drug resistance in cutaneous leishmaniasis. Sb^V^ must be reduced to its trivalent active form (Sb^III^). This reduction occurs within the host macrophage, and the resultant Sb^III^enters amastigotes via the aquaglyceroporin1 (AQP1) membrane carrier. Overexpression of AQP1 results in hypersensitivity of the parasites to Sb^III^, but resistant phenotypes accompany reduced expression, inactivation mutations, or deletion of AQP1. Hence, in this study, a phylogenetic analysis using barcode gene *COX*II and kDNA minicircle and expression analysis of *AQP1* were performed in treatment failure isolates to assess the isolates’ molecular characteristics and to verify possible association with drug response.

**Methods:**

Samples in this study were collected from patients with cutaneous leishmaniasis referred to the Diagnosis Laboratory Center in Isfahan Province, Iran, from October 2017 to December 2019. Among them, five isolates (code numbers 1–5) were categorized as treatment failures. The PCR amplification of barcode gene COXII and kDNA minicircle were done and subsequently analyzed using MEGA (10.0.5) to perform phylogenetics analysis of Treatment failures (TF) and Treatment response (TR) samples. Relative quantification of the AQP1 gene expression of TF and TR samples was assessed by real-time PCR.

**Results:**

All samples were classified as *L. major*. No amplification failure was observed in the cases of barcode gene *COX*II and kDNA minicircle amplification. Having excluded the sequences with complete homology using maximum parsimony with the Bootstrap 500 method, four major groups were detected to perform phylogenetic analysis using *COX*II. The phylogenetic analysis using the barcode target of minicircle showed that all five treatment failure isolates were grouped in a separate sub-clade.

**Conclusions:**

We concluded that the barcode gene *COX*II and the minicircle kDNA were suitable for identification, differentiation and phylogenetic analysis in treatment failure clinical isolates of *Leishmania major*. Also, *AQP1* gene expression analyses showed that treatment failure isolates had less expression than TR isolates. The isolate with TF and overexpression of the *AQP1* gene of other molecular mechanisms such as overexpression of ATP-binding cassette may be involved in the TR, such as overexpression of ATP-binding cassette which requires further research.

## Introduction

Leishmaniasis is a common disease in the tropical and subtropical regions of the world with a wide range of clinical symptoms caused by *Leishmania* spp. and transmitted by *Phlebotomus* spp. The symptoms vary from spontaneous cutaneous lesions to lethal visceral forms (Burza, Croft & Boelaert, 2018). Cutaneous Leishmaniasis (CL) is considered the most common form of this disease. Based on the World Health Organization (WHO) report, leishmaniasis is listed among 20 neglected tropical diseases (World Health Organization, 2018a). According to the WHO, annually, more than 350 million people in over 98 countries are exposed to new infections, which are more than 2 million. Cutaneous leishmaniasis involved roughly 0.6 to 1 million cases (Burza, Croft & Boelaert, 2018; Mouttaki et al., 2014; Tabbabi, 2019). CL is endemic in various parts of Europe, Africa, and Asia, particularly in Central Asia and the Middle East. Many new CL cases have been reported from six countries, including Afghanistan, Algeria, Brazil, Colombia, the Syrian Arab Republic and the Islamic Republic of Iran, in 2015. It is assumed that many factors, including environmental and individual migration, travel, increased immunosuppressed patients, and inappropriate insecticides, are contributing factors to the higher prevalence of new CL cases (Centers for Disease Control & Prevention, 2018; World Health Organization, 2018b). The CL epidemic was reported in the Syrian Arab Republic, followed by out-breaks in Lebanon, Jordan, and Turkey. Besides, there have been some reports from the outbreaks in Iran, Iraq, and Colombia (Alasaad, 2013; Alawieh et al., 2014; Du et al., 2016; Hayani, Dandashli & Weisshaar, 2015; Inci et al., 2015). The CL outbreaks may be due to the progressive and closer contacts of human beings with sandflies, human population expansion and moving to suburb areas where sandflies and reservoirs inhabit, or due to exposure with the hosts in the endemic areas (Magill, 2015). In some other endemic areas, such as Texas in the South Central Region of the United States, travel has been considered main reason (Bravo, 2018; Wright et al., 2008). CL is prevalent in many parts of Iran such as Tehran, Mashhad, Tabriz, Kerman, Isfahan, Neyshabur, Bam and Yazd. CL occurred in urban and rural regions is mainly caused by *L. tropica* and *L. major*, respectively.

Initially, *Leishmania* detection was based on the size, the shape of the wound, geographic location, and the injections to the laboratory animals (Fotouhi-Ardakani et al., 2016). These methods could not accurately identify the parasite species and strains, so molecular techniques were used as a suitable alternative method (Marcili et al., 2014). Molecular techniques also help to determine the parasite’s genetic characterization, which can be considered to design precise and appropriate control and preventive programs and develop an efficient vaccine and drug (Khosravi et al., 2012). Various target genes were used from mitochondria and nucleus (Stevenson, Fedorko & Zelazny, 2010; Lopes et al., 2016) used in a phylogenetic study of *Leishmania*, including, internal transcribed spacer of ribosomal DNA (ITS-rDNA; Hijawi, Hijjawi & Ibbini, 2019), heat shock proteins (Espada et al., 2018), cytochrome b (Yuan et al., 2017), kinetoplast DNA (kDNA; Dalimi et al., 2018.), 7SL RNA gene (Razavinasab et al., 2019), and cytochrome oxidase II (COXII; Cao et al., 2011). It is essential to use valuable gene targets as the barcode genes to show CL agents’ different characterization (Ziaei Hezarjaribi et al., 2020).

To control CL, WHO developed standardized tools for collecting of indicators such as the status of endemicity of CL, the number of reported CL cases, and the number of imported CL cases. One of the important strategies for controlling the disease is the treatment method. Various treatment methods were reported, including physical (Hejazi, Eslami & Dalimi, 2004; Gonçalves & Costa, 2018) and chemical treatment (Chakravarty & Sundar, 2019). Recently, some treatment failure cases (TF) have been reported from different parts of the world (Ahmadian et al., 2019; Alijani et al., 2019; Eslami et al., 2016; Vieira-Gonçalves et al., 2019). It seems that molecular characterization and phylogenetic studies of TF isolates can help find suitable strategies to design control programs and develop vaccines and drugs.

Treatment failures is one of the most critical factors, environmental changes, and host immune status leishmaniasis’ epidemiology as a re-emerging disease. One of the most important and well-known adaptation factors for the parasite against antimoniate is the aquaglyceroporin1 (AQP1, Mandal et al., 2010) protein encoded by *AQP1* gene. AQP1 is a member of the aquaporin superfamily, a membrane channel, that its role is the transportation of trivalent antimony (Sb^III^) inside the parasite (Gourbal et al., 2004). Overexpression of *AQP1* in *L. major* has been reported in treatment response (TR) isolates in many studies (Gourbal et al., 2004; Richard et al., 2004; Marquis et al., 2005).

In this study, the TF clinical isolates obtained from patients with CL were characterized using phylogenetic analysis by the barcode gene *Cytochrome oxidase* II (*COX*II) and the conserved region of kDNA minicircle. Also, the *AQP1* gene expression was assessed in TF isolates using SYBR Green quantitative real-time PCR. The GAPDH (Glyceraldehyde-3-Phosphate Dehydrogenase) was used as the endogenous control.

## Materials and Methods

### Ethical statement

The current study was approved by the Ethics Committee of Shahid Sadoughi University of Medical Sciences, Yazd, Iran (Approval ID IR.SSU.MEDICINE.REC.1396.323). All patients who participated in this study signed a written informed consent form before sampling.

### Population study

In this study, the main target population was the patients referred to the Diagnosis Laboratory Center in Isfahan Province, Iran, from October 2017 to December 2019 and diagnosed with CL but categorized as TF. The inclusion criteria were CL patients failed to be treated with the standard course of glucantime regimen, without any interruption during the treatment, previous anti-*Leishmania* treatment except for glucantime, and anti-*Leishmania* co-therapy. Some treatment-responsive isolates were considered as the standard samples to compare the data.

The main objective of the research was to assess at molecular level the isolated from treatment failure prior to treatment of CL, to screen the selected marker of interest. The patients were treated with the standard regimen of glucantime. Response to the treatment was defined as re-epithelialization of lesions and decreased inflamed borders of the lesions. The patients with lesions and without a response at the end of the complete standard treatment course were considered treatment failures (TF, Martínez et al., 2020; Vanaerschot et al., 2014). The cases who responded to glucantime were considered as TR.

### Sampling

Samples were collected by scrubbing the lesion edge after disinfecting with 70% alcohol. Two slides were prepared for either direct microscopic examination or molecular analysis for each patient. The biopsy was taken from the edges of lesion skin, transferred into RNA*later* solution (Ambion, Inc., Austin, TX, USA), and then stored at −20 °C until the use.

### Detection and identification of the isolates

A microscopic examination was carried out to find the Leishman body. The DNA was extracted from smears using the DNA extraction kit (GeneAll, South Korea) based on the manufacturer’s instructions. The quantity of isolated DNA was evaluated using nanodrop (ABI, Vernon, CA, USA). To detect the *Leishmania* genus, ITS1-PCR was performed using the primer pair of LITSr-F and L5.8s-R (Eslami et al., 2012). Each amplification reaction mixture was included 1 X PCR buffer, 0.2 mM dNTP, 1.5 mM MgCl_2_, 1.5 U *Taq* DNA polymerase, 0.5 µM for each primer, and 100 ng DNA. The thermal profile of PCR amplification was comprised of the first denaturation at 94 °C for 5 min, followed by 35 cycles of denaturation at 94 °C for 45 s, annealing at 50 °C for 45 s, and elongation at 72 °C for 45 s. The final elongation was done at 72 °C for 5 min. Positive and negative controls were included in each run using DNA extracted from *L. major* (MRHO/IR/75/ER) and ddH_2_O, respectively. The amplification product was analyzed using 1% agarose gel electrophoresis. The expected amplicons size was about 300–350 bp for the verification of *Leishmania* spp. Subsequently, the amplification products were digested with *Hae* III (*Bsu* RI) restriction endonuclease enzyme for three h at 37 °C; heat inactivation was done and then analyzed on 3% agarose gel. Digestion product produced two main bands of 220 and 140 bp was indicative of *L. major* species. *L. major* (MRHO/IR/75/ER) was included as a positive control. All tests were done in triplicate.

### Molecular analysis by *COX*II

To amplify *COX*II, the primer pair of COXII-F 5′-GCATAAATCCATGTAAAACACCACA-3′ and COXII-R 5′-TGGCTTTTATWTTATCATTTTGAATG-3′ was used (Oliveira et al., 2011). Each amplification reaction mixture was included 1 X PCR buffer, 0.2 mM dNTP, 1.5 mM MgCl_2_, 1.5 U *Taq* DNA polymerase, 0.5 µM for each primer, and 100 ng DNA. The thermal profile of PCR amplification was compromised of the first denaturation at 94 °C, followed by 35 cycles of denaturation at 94 °C for 60 s, annealing at 57 °C for 20 s, and elongation at 72 °C for 30 s. The final elongation was done at 72 °C for 5 min. The positive and negative controls were included in each run using DNA extracted from *L. major* (MRHO/IR/75/ER) and ddH_2_O, respectively. The 1.5% agarose gel electrophoresis was used for the amplification product. The expected amplicon size was 602 bp.

### Molecular analysis by kDNA minicircle

Molecular analysis of kDNA minicircle was performed as described previously (Aghai-Maybodi et al., 2018). Positive and negative controls were included in each run as *L. major* (MRHO/IR/75/ER) and ddH_2_O, respectively. Amplicon size of 120 bp was considered as positive results.

### Sequencing and molecular analysis

PCR amplifies fragments of *COX*II in all 5 TF were subjected to direct sequencing. Sixteen treatment responsive isolates were also sequenced for the sake of comparison between TF and TR isolates. The same has been done for kDNA minicircle amplicons. The repairing and then BLAST for all sequences were done. All the mentioned sequences were submitted in the GenBank, NCBI with the accession numbers from MK972457 to MK972460. Similar sequences were searched using BLASTn (Altschul et al., 1997). The included sequences are in Table 1. Multiple alignments using T-COFFE were done (Notredame, Higgins & Heringa, 2000). The software of MEGA 10.0.5 was used for phylogenetic analysis (Kumar, Stecher & Tamura, 2016). The evolutionary history was inferred using the maximum parsimony method with Bootstrap 500. This method showed the most Bootstrap.

**Table 1:** The retrieved sequences related to the barcode gene *COX*II used in this study.

Accession number	Definition
MH443402.2	*Leishmania major* isolate Varzaneh-114 cytochrome oxidase subunit II (COII) gene, partial sequence; kinetoplast
MK972457.1	*Leishmania major* isolate Yazd-1 cytochrome oxidase subunit II (COII) gene, partial sequence; kinetoplast
MH443393.2	*Leishmania major* isolate Varzaneh-100 cytochrome oxidase subunit II (COII) gene, partial sequence; kinetoplast
MK972459.1	*Leishmania major* isolate Yazd-3 cytochrome oxidase subunit II (COII) gene, partial sequence; kinetoplast
MH443400.2	*Leishmania major* isolate Varzaneh-111 cytochrome oxidase subunit II (COII) gene, partial sequence; kinetoplast.
MH443404.2	*Leishmania major* isolate Varzaneh-116 cytochrome oxidase subunit II (COII) gene, partial sequence; kinetoplast
MH443399.2	*Leishmania major* isolate Varzaneh-110 cytochrome oxidase subunit II(COII) gene, partial sequence; kinetoplast
MH443397.2	*Leishmania major* isolate Varzaneh-106 cytochrome oxidase subunit II(COII) gene, partial sequence; kinetoplast
MH443396.2	*Leishmania major* isolate Varzaneh-105 cytochrome oxidase subunit II (COII) gene, partial sequence; kinetoplast
MH443391.2	*Leishmania major* isolate Varzaneh-98 cytochrome oxidase subunit II (COII) gene, partial sequence; kinetoplast
MH443398.2	*Leishmania major* isolate Varzaneh-109 cytochrome oxidase subunit II (COII) gene, partial sequence; kinetoplast
MH443394.2	*Leishmania major* isolate Varzaneh-102 cytochrome oxidase subunit II (COII) gene, partial sequence; kinetoplast
MH443403.2	*Leishmania major* isolate Varzaneh-115 cytochrome oxidase subunit II (COII) gene, partial sequence; kinetoplast
MH443390.2	*Leishmania major* isolate Varzaneh-97 cytochrome oxidase subunit II (COII) gene, partial sequence; kinetoplast
MH443401.2	*Leishmania major* isolate Varzaneh-113 cytochrome oxidase subunit II (COII) gene, partial sequence; kinetoplast
MH443389.2	*Leishmania major* isolate Varzaneh-96 cytochrome oxidase subunit II (COII) gene, partial sequence; kinetoplast
MK972460.1	*Leishmania major* isolate Yazd-4 cytochrome oxidase subunit II (COII) gene, partial sequence; kinetoplast
MK972461.1	*Leishmania major* isolate Yazd-5 cytochrome oxidase subunit II (COII) gene, partial sequence; kinetoplast
MH443395.2	*Leishmania major* isolate Varzaneh-104 cytochrome oxidase subunit II (COII) gene, partial sequence; kinetoplast
MK972458.1	*Leishmania major* isolate Yazd-2 cytochrome oxidase subunit II (COII) gene, partial sequence; kinetoplast
MH443388.2	*Leishmania major* isolate Varzaneh-63 cytochrome oxidase subunit II (COII) gene, partial sequence; kinetoplast
KF815208	*Leishmania major* isolate 110 clone 6 cytochrome oxidase subunit II (COII) gene, partial sequence, kinetoplast
KY407539	*Leishmania major* isolate IMP/Fars1 cytochrome oxidase subunit II (COXII) gene, partial sequence, kinetoplast
KU680818	*Leishmania major* strain MHOM/SU/73/5ASKH cytochrome oxidase subunit II (COII) gene, partial sequence, kinetoplast
KU680819	*Leishmania major* strain MRHO/IR/75/ER cytochrome oxidase subunit II (COII) gene, partial sequence, kinetoplast
KF815210	*Leishmania major* isolate 109 clone 3 cytochrome oxidase subunit II (COII) gene, partial sequence, kinetoplast
KF815211	*Leishmania major* isolate 109 clone 2 cytochrome oxidase subunit II (COII) gene, partial sequence, kinetoplast
EU140338	*Leishmania major* isolate MHOM/SU/73/5ASKH maxicircle, partial sequence, mitochondrial
AF287688	*Leishmania major* isolate MRJX cytochrome c oxidase subunit II (COII) gene, partial sequence, kinetoplast gene for kinetoplas product
EF633106	*Leishmania major* cytochrome oxidase subunit II (COII) gene, complete sequence, mitochondrial
KU680820	*Leishmania major* strain MHOM/IL/80/Friedlin cytochrome oxidase subunit II (COII) gene, partial sequence, kinetoplast

### RNA extraction and cDNA synthesis

Total RNA was extracted from all TF samples using the RNeasy Plus Mini Kit (Qiagen, HildenGermany). Five TR samples were also analyzed as standard control isolates. The RNA quality and quantity were analyzed using 1% agarose gel electrophoresis and spectrophotometer (Eppendorf BioPhotometer Plus, Eppendorf, Germany), respectively. Then, cDNA was synthesized using high capacity cDNA reverse transcription kit (Applied Biosystems, Foster City, CA, USA) with oligo dT and random hexamer primers based on the manufacturer’s instruction.

### *AQP1* expression analysis

*AQP1* gene expression analysis was performed as described previously (Eslami et al., 2016). Thermal cycling profile applies as follows; initial denaturation at 95 °C for 10 min followed by 40 cycles of denaturation at 95 °C for 10 s, and annealing and extension at 60 °C for 20 s. The melting curve analysis verified the specificity of the real-time PCR reaction. The relative expression (RQ) analysis was done using the following formula:

\eqalignb{2^{\Delta \Delta {\rm Ct}}= ({\rm Ct}_{\text{target\ gene\ of\ TF(AQP1)}}-{\rm Ct}_{\text{reference\ gene\ of\ TF(GAPDH)}})- ({\rm Ct}_{\text{target\ gene\ of\ TR(AQP1)}}\cr-{\rm Ct}_{\text{reference\ gene\ of\ TR(GAPDH)}})

### Statistical analysis

The statistical analysis was done using the non-parametric Mann–Whitney *U* test for *AQP1* gene expression between two groups of TF and TR isolates.

## Results

### Detection and identification of the isolates

Molecular identification of the isolates using PCR-RFLP produced fragments of 300–350 bp, hence *Leishmania* spp. was confirmed. RFLP analysis was performed by *Hae* III restriction enzyme, which produced 220 and 140 bp fragments indicating *L. major*.

### Molecular analysis and phylogenetic analysis by *COX*II

All TF isolates were successfully amplified using the primer pair of *COX*II (Supplemental File 1). The sequencings were successfully performed, and all of them were submitted at GenBank, NCBI, from MK972457 to MK972461.

Sequences of the *COX*II region from TF isolates were analyzed using nBLAST, T-COFFEE, multiple alignments, and MEGA (10.0.5) software. The multiple alignments of five TF isolates’ sequences were done with the Friedlin strain of *L. major* (Supplemental File 2). Accordingly, all five TF isolates had conserved areas in most sites compared to the European *L. major* (Friedlin), being significantly different from the standard European strain in several nucleotides except in terminal regions. The TF isolate with code 2 differed from the European strain with the dispersed point in two nucleotides, even though, all TF isolates were dissimilar with the European standard strain in four nucleotides (Supplemental File 2).

**Figure 1:**
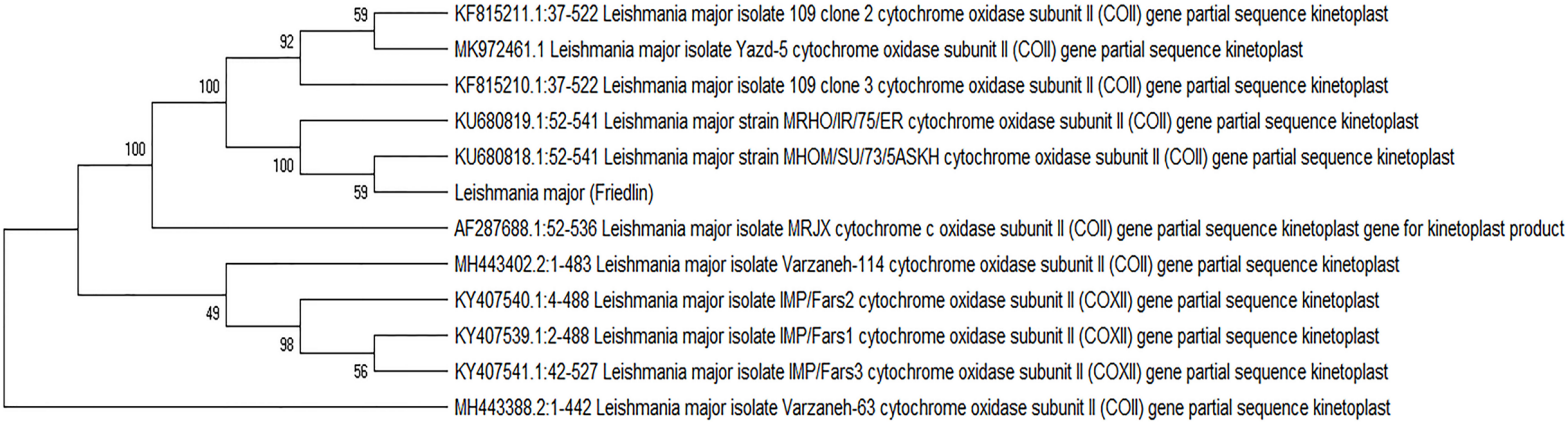
Phylogenetic tree summarizing the relationship among the *COX*II sequences of *Leishmania* species with various responses to treatment using the maximum parsimony method with Bootstrap 500. Phylogenetic tree of the *COXII* gene sequence of 21 isolates with previously known isolates (GenBank KF815208.1, KY407539.1, KU680818.1, KU680819.1, KF815210.1, KF815211.1, EU140338.1, AF287688.1 and EF633106.1) shows distinct clusters.

Sequences with complete homology were excluded, leaving only one representative to draw a phylogenetic tree using the barcode gene *COX*II. In Fig. 1, there are four major groups. The first group consists of only MH44388 *Leishmania* Varzaneh-63, while the second group consists of two clades. The first clade contains two sub-clades, and the first sub-clade consists of KY407541.1 *Leishmania* IMP/FARS3 and KY407539 *Leishmania* IMP/FARS1, and the second sub-clade contains KY407540 *Leishmania* IMP/FARS2. The second clade includes MH443402 *Leishmania* Varzaneh-114, when the third group includes only AF287688 *Leishmania* MRJX. The fourth group consists of 2 clades, and the first clade is divided into two sub-clades. The first sub clade contains *Leishmania* major and KU680818 *Leishmania* MHOM/SU/73/5ASKH. The second sub-clade is KU680818 *Leishmania* MRHO/IR/75/ER, and the second clade contains two sub-clades. The first sub-clade contains KF815210 *Leishmania* isolates 109 clone3, and the second contains MK972461 *Leishmania* Yazd 5 and KF815211 *Leishmania* isolates 9 (Fig. 1).

### Molecular analysis and phylogenetic analysis by kDNA minicircle

**Figure 2:**
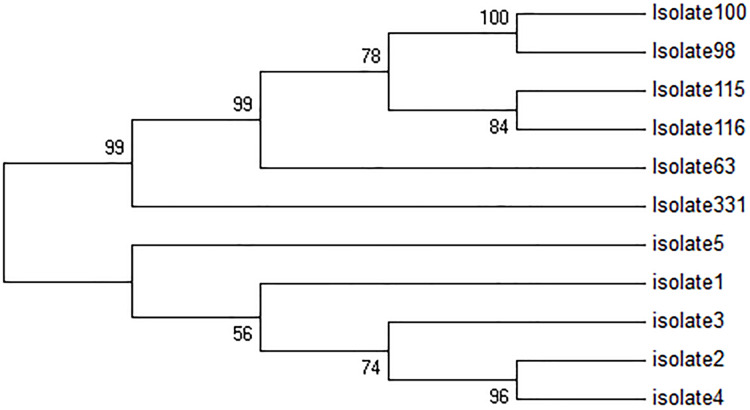
Phylogenetic tree summarizing the relationship among the kDNA minicircle sequences of *Leishmania* species with various responses to treatment using the maximum parsimony method with Bootstrap 50. The phylogenetic tree shows separation of the treatment failure (TF) and treatment response isolates.

All five TF isolates were assessed, and none of the sequences related to kDNA minicircle were deposited in GenBank, NCBI. In Fig. 2, four major groups are shown. The first group consisted of two clades, while the first clade contains two sub-clades. The first sub-clade contained isolates 4, 2 and second isolate 3. The second clade contained isolate 1. Besides, isolates 2 and 4 were very similar. The second group only consisted of isolate 5, and the third group included only isolate 331. The fourth group consisted of 2 clades. The first clade was isolate 63, and the second clade was divided into two sub-clades, isolates 115 and 116, and isolates 98 and 100. In this group, isolates 98 and 100 had high similarity with each other.

### The gene expression of AQP1

**Figure 3:**
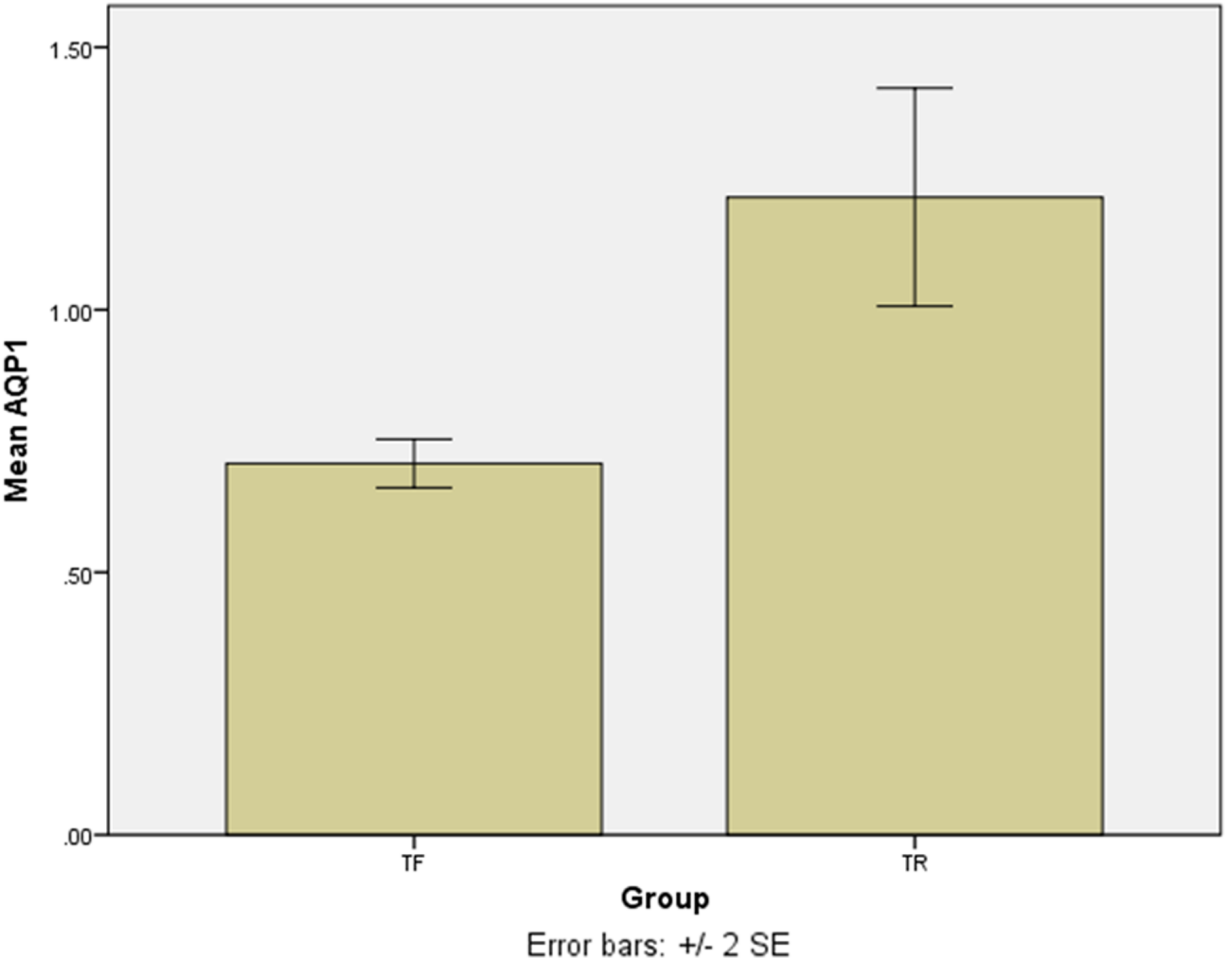
The mean score of *AQP1* gene expression in treatment failure (TF) isolates and treatment response (TR) isolate. The *AQP1* gene expression in most treatment failure (TF) isolates is lower comparing to the mean gene expression in TR isolates.

The SYBR Green real-time PCR showed that one isolate had the overexpression among the TF isolates with a mean score of 2.75 ± 0.05. Except for the last isolate, the mean gene expression of *AQP1* in 4 TF isolates was 0.7 ± 0.04 (Fig. 3). The difference was statistically significant (*p* = 0.016). The results of the *AQP1* gene expression is detailed in Table 2.

**Table 2:** The Fold change of *AQP1* gene expression in comparison with the TR1 as the standard sample.

Isolates	Mean ± standard deviation
TF*1	0.669595 ± 0.06
TF2	2.75474 ± 0.05
TF3	0.666 ± 0.04
TF4	0.74118 ± 0.03
TF5	0.753483 ± 0.04
TR*1	1
TR2	1.195087 ± 0.03
TR3	1.462111 ± 0.05
TR4	1.439211 ± 0.04
TR5	0.974696 ± 0.10

**Note:**

* TF, treatment failure; TR, treatment response.

## Discussion

This study evaluated the molecular characterization of TF clinical isolates of *Leishmania major* obtained from patients diagnosed with CL. The molecular identification showed that all isolates were *L. major*. Hence, all of the patients were treated with the standard regimen of glucantime and regularly checked by a physician. The phylogenetic analysis was studied using the barcode gene *COX*II and the kDNA minicircle.

The sequences of *COX*II were submitted at GenBank, NCBI. Cytochromes are considered one of the most useful genes for molecular and phylogenetic studies (Landis & Koch, 1977; Ibrahim & Barker, 2001). The *COX*II has been shown a higher evolutionary rate in some other studies (Cao et al., 2011; Lopes et al., 2016). Because of its multicopy nature, it can be used as clinical material and environmental samples. Nevertheless, only a few phylogenetic studies of the genus Leishmania have included this marker and its discriminative power has not been established in detail (Ibrahim & Barker, 2001; Cao et al., 2011; Lopes et al., 2016). The present study showed that it could be useful for the discrimination of subgenera or species complexes, but its applicability for species delimitation needs further research. In our study, the region of *COX*II was used for amplification and phylogenetic analysis (Lopes et al., 2016).

The barcode gene *COX*II application showed satisfactory confirmatory outcome for *Leishmania* and *L. major* detection of these isolates. Isolates had many conserved sites with the European standard *L. major*. Except in the terminal regions, all five TF isolates were significantly different from the European strain of *L. major* (Friedlin) in several nucleotides. Multiple alignment analysis disclosed that these isolates contained many central conserved areas. We agree with the previous study (Cao et al., 2011) that the *COXII* barcode can distinguish genus and species. There are several studies on phylogeny analysis using *cytochrome b* and *COXI* (Mohammadpour et al., 2019; Yuan et al., 2017), but limited study is available about *COX*II phylogeny analysis for *Leishmania* spp. It seems that a large sample size analysis would be necessary to discover more roles for this region. This region is used to differentiate strains in different geographical regions (Martínez et al., 2010).

The kDNA minicircle region could differentiate TF and TR isolates successfully (Fig. 2). It seems that the kDNA minicircle could be considered as one of the important regions for the differentiation of isolates with different phenotypes. This result is in agreement with the finding reported by Aghai-Maybodi et al. (2018). We suggest using this area for the differentiation of TF isolates in some other different geographical areas.

The treatment failure has been studied extensively in *Leishmania* spp. especially in *L. major*. It is already known that pentavalent antimony (Sb^V^) gets converted to trivalent form (Sb^III^) inside the macrophage by a putative metalloid reductase (Sereno et al., 1998). TF isolates may be influenced by increased expression of the molecules in thiol biosynthetic pathway (Fairlamb & Cerami, 1992; Haimeur et al., 1999), overexpression of the ABC transporter with sequestering SbIII–thiol conjugate (Legare et al., 2001; Mukherjee et al., 2007) and low expression of the *AQP1* gene, and decreasing the concentration of antimonial inside the parasite (Gourbal et al., 2004; Marquis et al., 2005; Maharjan et al., 2008).

In this study, the *AQP1* gene expression, which is the important gene encoding AQP1 molecule, and is significant in entering antimoniate to the parasite, was assessed. The results showed that TF isolates had low expression of *AQP1* in comparison with TR isolates. However, one of the TF isolates showed the overexpression of *AQP1*.

Failure of antimoniate treatment in *L. major* infected patients was detected to be related to the disruption of at least one of the alleles of *AQP1* (Gourbal et al., 2004; Marquis et al., 2005). Our previous study showed the overexpression of *AQP1* gene in TF clinical isolates of *L. major* (Eslami et al., 2016). Furthermore, another studies showed mutation in *AQP1* in both TF and TR clinical *L. major* isolates (Alijani et al., 2019; Eslami et al., 2019). Any mutation, deletion, and insertion in *AQP1* gene can change the frame reading and therefore produce a non-functional protein (Potvin et al., 2020). Therefore, overexpression of *AQP1* gene can be seen but the protein has no function. In our study, TF2 had overexpression of *AQP1* but it showed failure in treatment. This, more researches are required to find the mechanisms of failure in treatment in this particular isolate.

## Conclusions

This study indicates that the *COX*II region and kDNA minicircle were suitable for the identification, differentiation, and phylogenetic analysis in treatment failure of clinical isolates of *Leishmania major*. The *AQP1* gene expression revealed that the other molecular mechanisms might involve the treatment failure in one of the clinical isolates studied in the present study, such as the overexpression of ATP-binding cassette transporters to influence the efflux of drugs.

## Supplemental Information

10.7717/peerj.10969/supp-1 Supplemental Information 1 Agarose gel electrophoresis for amplicons analysis of COXII region. Lane 1: 50 bp DNA ladder, lane 2: Leishmania major (MRHO/IR/75/ER) as positive control, lane 2: TF isolate, lane 3: TR isolate, lane 4: negative control. The expected fragment size was 60.

10.7717/peerj.10969/supp-2 Supplemental Information 2 Multiple alignment of COXII sequence for 5 TF isolates with the same sequence from the standard strain of Leishmania major (Friedlin) using T-COFFE. Isolates 1 to five are the treatment failure isolates and "Leishmania" is the European standard strain of.

10.7717/peerj.10969/supp-3 Supplemental Information 3 Sequence alignment of the isolates.

10.7717/peerj.10969/supp-4 Supplemental Information 4 Sequences of Leishmania major COII isolates. Amino acid sequences of Leishmania major cytochrome oxidase subunit II (COII) gene isolates.

10.7717/peerj.10969/supp-5 Supplemental Information 5 PCR sequencing data (for Fig. 3). Raw PCR data supporting the results shown in Fig. 3.

10.7717/peerj.10969/supp-6 Supplemental Information 6 Additional anonymous patients’ data.

## Additional Information and Declarations

### DNA Deposition

The following information was supplied regarding the deposition of DNA sequences: The COI gene sequences are available at GenBank: MK972457, MK972458, MK972459 and MK972460.

### Data Availability

The following information was supplied regarding data availability: Data is available in the Supplemental Files.
