# Supplementary figures and images for: Molecular characteristic of treatment failure clinical isolates of Leishmania major
Source: PeerJ. 2021 Mar 11;9:e10969. doi: 10.7717/peerj.10969 (PMC7956003; doi:10.7717/peerj.10969)

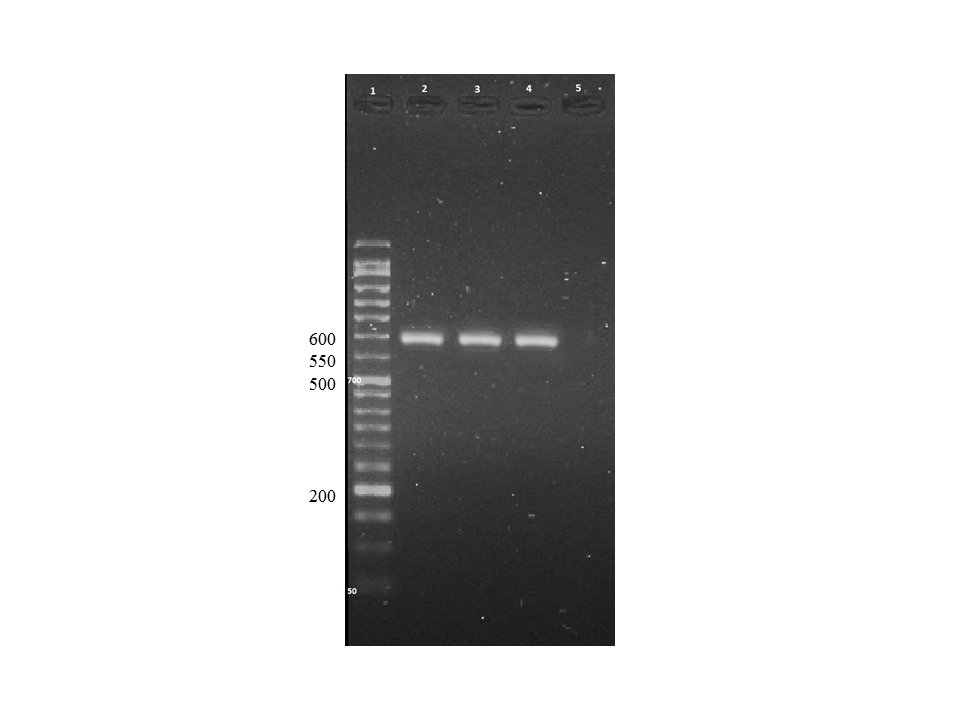

Supplement: Supplemental Information 1 [file peerj-09-10969-s001.png]

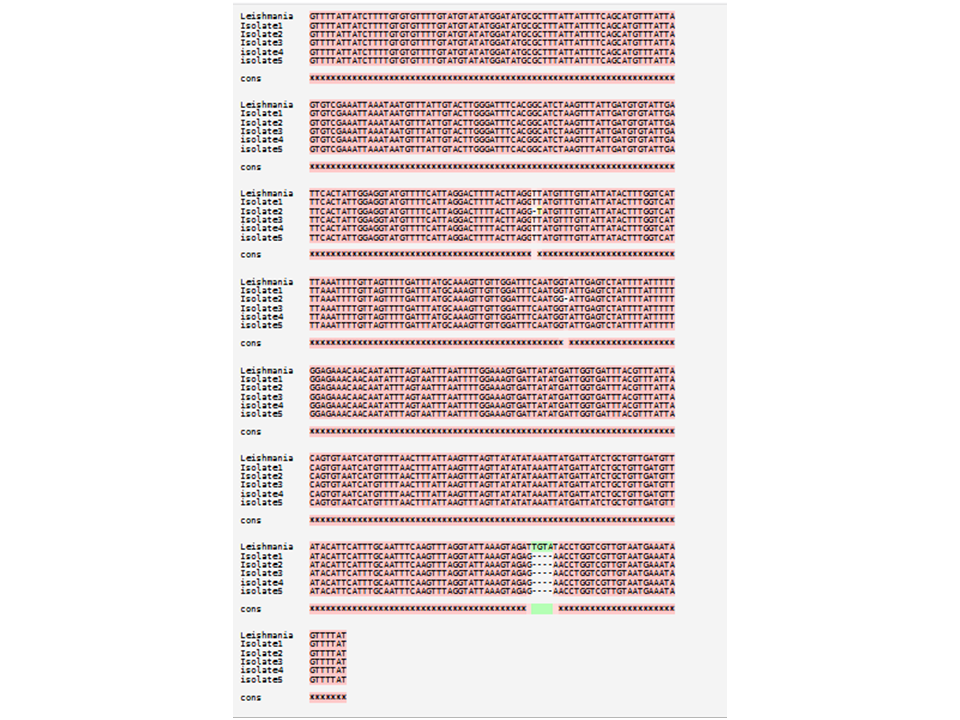

Supplement: Supplemental Information 2 [file peerj-09-10969-s002.png]
